# Time, money, and weight loss: a qualitative study exploring patients’ perspectives on randomization for bariatric surgery vs. an intensive non-surgical weight loss program

**DOI:** 10.1186/s13063-025-08816-8

**Published:** 2025-04-04

**Authors:** Sofie Amalie Tomova-Olsen, Marius Brostrøm Kousgaard, Katrine Tranberg Jensen, Susanne Reventlow, Ann-Kathrin Lindahl Christiansen, Kirstine Nyvold Bojsen-Møller, Carsten Dirksen, Gritt Overbeck

**Affiliations:** 1 Centre for General Practice, the Research Unit for General Practice and Section of General Practice, Department of Public Health, University of Copenhagen, Copenhagen, Denmark; 2 Department of Respiratory Diseases and Endocrinology, Copenhagen University Hospital Hvidovre, Hvidovre, Denmark; 3 Department of Clinical Medicine, University of Copenhagen, Copenhagen, Denmark

**Keywords:** Recruitment, Randomized controlled trial, Obesity, Bariatric surgery, Qualitative research

## Abstract

**Background:**

Randomized controlled trials (RCTs) are foundational in advancing medical knowledge and patient care, offering high-quality evidence on the comparative effectiveness of healthcare interventions. However, a common challenge for RCTs is the recruitment of trial participants. To understand and overcome potential obstacles in recruitment for a clinical trial (the LightBAR trial, NCT06309238) comparing the effectiveness of bariatric surgery versus an intensive weight loss program, a qualitative study was conducted.

**Methods:**

Nine patients from the public bariatric surgery waiting list participated in focus groups at a hospital in the Capital Region of Denmark. Vignette scenarios were utilized to prompt participants to reflect on barriers and facilitators for participation. Three patients participated in a follow-up interview. Data was analyzed using thematic analysis.

**Results:**

Analysis revealed four main themes: (1) having waited long for surgery reduced participants’ willingness to be randomized; (2) the cost of weight loss medication was a major concern for participants; (3) participants were concerned about the extra work involved in program participation; and (4) participants weighed the efficacy and potential negative side effects of surgery against those of an intensive weight loss program based on personal beliefs and experiences.

**Conclusions:**

Tailoring the recruitment strategy to patients’ circumstances and concerns, and providing clear, patient-centered communication about the nature and potential implications of participating in the trial may improve recruitment success.

**Trial registration:**

The LightBAR trial (NCT06309238). Registered on ClinicalTrials.gov on May 2, 2024.

**Supplementary Information:**

The online version contains supplementary material available at 10.1186/s13063-025-08816-8.

## Background

Clinical trials, particularly randomized controlled trials (RCTs), are pivotal in advancing medical knowledge and patient care. However, conducting RCTs in which intervention and comparator are fundamentally different poses unique challenges, e.g., when participants are randomized to a surgical intervention such as bariatric surgery versus non-surgical treatment [1]. These challenges predominantly revolve around recruitment and retention [2]. Different models address these complexities, emphasizing tailored approaches to enhance recruitment and retention, including refining eligibility assessments and conveying clinical equipoise to optimize recruitment efforts [3]. Moreover, the recruitment process is multifaceted, ranging from identifying potentially eligible patients to obtaining informed consent.

The employment of qualitative research before trials has emerged as a valuable strategy for understanding and tackling recruitment difficulties [4]. This approach leverages qualitative methods to gain insights into the complex social, behavioral, and cultural factors influencing recruitment and then uses these insights to adjust trials to improve recruitment and retention outcomes [5, 6]. However, it has been stressed in the literature that qualitative work investigating recruitment and retention pretrial should focus not only on the barriers but also on the facilitators and make suggestions for potential changes in future strategies [7].

Previous studies have investigated the recruitment of participants for surgical interventions and found barriers to obtaining the number of participants needed to power RCTs [8, 9]. However, to our knowledge, no studies have looked into the recruitment of participants from a bariatric waiting list for alternative intensive weight loss interventions.

This study is nested in the preparatory work for LightBAR, an RCT comparing an intensive weight loss intervention (IWL) to bariatric surgery for individuals with severe obesity. Understanding recruitment dynamics in this specific context is crucial to ensuring trial feasibility and evaluating whether IWL can serve as a viable, less invasive alternative to surgery. This study adds new insights into recruitment challenges for RCTs comparing surgical and non-surgical weight loss treatments. It is, to our knowledge, the first study to explore how patients on a bariatric surgery waiting list perceive randomization, informing future trial recruitment strategies.

## Methods

### Setting

The LightBAR trial (NCT06309238) is an RCT that will compare the efficacy and safety of an intensive weight loss program compared with bariatric surgery. The intensive weight loss program is a dietitian-led 104-week intervention that includes twelve weeks of total diet replacement (a low-calorie diet of approximately 800 kcal/day consisting of powdered shakes and meals) followed by an energy-reduced healthy diet and increased physical activity and may be combined with the use of weight loss medication. The trial is conducted in Denmark and the United Kingdom and will run from 2024 to 2027. In Denmark, participants fulfilling national requirements for bariatric surgery [10] will be recruited after referral to three of five public bariatric surgery centers and asked if they will be willing to participate in a clinical trial in which they will be randomized to either the intensive weight loss program (intervention) or bariatric surgery (comparator). Given the complexities of patient recruitment and retention in RCTs comparing surgical and medical treatments, particularly in the context of bariatric surgery [2], it was anticipated that recruitment for the LightBAR trial would be challenging. As a result, a qualitative study was conducted before the commencement of the LightBAR trial to explore factors influencing the willingness of patients on a public bariatric surgery waiting list to participate in a clinical trial that involves random assignment to either bariatric surgery or an intensive weight loss program. Understanding these factors may inform strategies to optimize recruitment communication and the timing of recruitment.

### Focus groups and individual follow-up interviews with patients

**Fig. 1:**
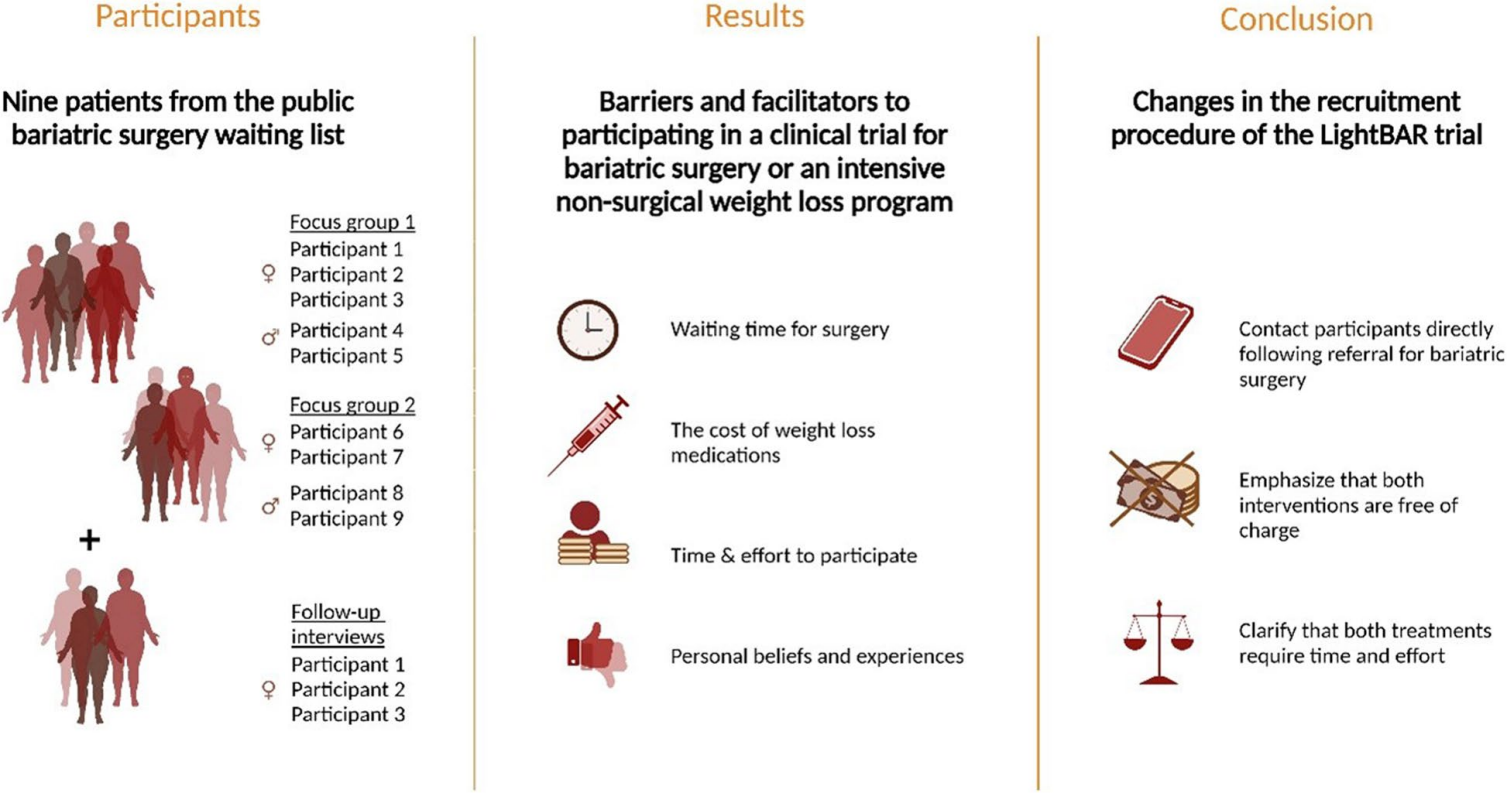
Overview of participants, themes, and conclusion. Figure created with BioRender.com

Focus groups were chosen as the primary method based on their ability to shed light on different perspectives among participants and facilitate dynamic discussions, making them ideal for exploratory studies [11]. In May 2023, there were 408 patients on the public bariatric surgery waiting list in the Capital Region of Denmark of which 30 patients attended one of two group information sessions in May and June 2023. Prior to the sessions, patients had been informed about the study by letter and were invited to participate in the focus groups during the information sessions. Patients were explicitly informed that their involvement in the focus groups was solely part of the preparatory work for LightBAR and would not lead to an invitation to participate in the trial. An endocrinologist from the hospital’s bariatric team (CD) provided general information about bariatric surgery at the information sessions and explained the core elements of the LightBAR trial before the start of the focus groups; The trial was presented as a randomized controlled trial comparing the effectiveness of bariatric surgery (active control arm) to an intervention consisting of total dietary replacement in combination with weight loss medication, increased physical activity, and behavioral support delivered by dietitians (intervention arm). Both treatments are provided free of charge. Participants were informed that the purpose of the focus groups was to identify potential barriers and facilitators to trial participation among patients on the bariatric waiting list. The two focus groups were conducted at the hospital immediately following the information sessions. There were five participants in the first focus group and four in the second (see Fig. 1 for participants). The focus groups were moderated by an experienced interviewer (GO), while a research assistant observed nonverbal communication.

Since the goal was to capture the thoughts and feelings of participants in a situation comparable to that of future participants, and since it was a premise that the participants in the focus groups would not themselves be part of the trial, we chose to use vignettes [12]. Additionally, vignettes [12] are suitable for exploring sensitive topics in a less personal and confrontational manner [13] and can be useful when exploring research topics where participants might be reluctant to share their personal experiences [14]. Participants in the focus groups were presented with two different vignettes depicting hypothetical individuals eligible for bariatric surgery. The vignettes (see Additional file 1) featured a single mother (Mette) in her 40 s with physically demanding work and a 60-year-old man (Peter) who desired to play soccer with his grandchildren. These individuals were offered participation in the trial in which they could be randomized to either an intervention group receiving an intensive non-surgical weight loss program (combining dietary replacement and weight loss medication), or to the bariatric surgery group, which served as the active control arm. The cases were intended to represent diversity in age, gender, and life circumstances. The participants in the focus groups were tasked with reflecting on the hypothetical cases outlined in the vignettes (see Additional file 2 for the moderator guide), which informed a discussion about the two weight loss treatments. At the end of the focus groups, participants were asked to share their contact information with the researcher (GO) if they wished to participate in an individual follow-up interview. The purpose of these individual interviews was to explore the topics discussed in the focus groups in more depth (see Additional file 3 for the interview guide). They complemented the focus groups by allowing participants to share more personal experiences and treatment preferences. Five of nine participants provided their contact information, however, two could not be reached, thus three (all female) participants took part in a follow-up interview. As a token of gratitude for their participation, participants received cinema gift cards worth approximately 200 DKK (27 EUR), which corresponded to the cost of two cinema tickets. The follow-up interviews were conducted over the phone by a research assistant, with no prior contact with participants. The follow-up interviews lasted between 30 and 60 min. The first focus group lasted 56 min, the second 25 min. Both sessions were video recorded to distinguish between participants during interview transcription. The interviews were conducted in Danish, with selected quotes translated into English afterwards. Although ethical approval is not a requirement under Danish law for performing interviews, all participants gave their written consent before the focus groups. Oral consent was obtained for telephone interviews to minimize participant burden. To enhance transparency in reporting the COREQ checklist [15] was used (see Additional file 4).

### Sample

Among the participants in the study, there was an equal distribution between men (four) and women (five). At the time of the study, the participants were in their 30 s to 50 s, residing in Greater Copenhagen, and were either studying or working. They had been on the public bariatric surgery waiting list for one to three and a half years, and the majority had previous experience with weight-loss medications.

#### Data analysis

Focus groups and individual interviews were transcribed and analyzed based on Braun and Clarke’s thematic analysis [16]. SO read the interviews several times, created initial codes, and organized them into themes in Microsoft Word. Extracted quotes and codes were discussed among five of the authors (SO, GO, MBK, KTJ, and SR) and re-coded by SO in an iterative process until four themes were conceptualized.

## Results

We identified the following four themes: (1) having waited long for surgery reduced participants’ willingness to be randomized; (2) the cost of weight loss medication was a major concern for participants; (3) participants were concerned about the extra work involved in program participation; and (4) participants weighed the efficacy and potential negative side effects of surgery against those of an intensive weight loss program based on personal beliefs and experiences.

### Having waited longer for surgery reduced participants’ willingness to be randomized

Participants had made multiple attempts to lose weight, achieving short-term success each time, only to regain the weight. This cycle led them to consider bariatric surgery as a last resort. By the time of the focus groups, they had already spent between one to three years on the public waiting list for bariatric surgery. This waiting period allowed them to mentally prepare for the procedure, making them apprehensive about waiting even longer if randomized to an intensive weight loss program that might not yield results.

Participants suggested that newly joined patients on the bariatric waiting list should first be invited to participate in the RCT, since they may be more receptive to exploring alternative options before committing to surgery. Some argued that patients nearing the age of 60 were more likely to undergo surgery due to the upper age limit for bariatric surgery, whereas younger patients may assume they have more time to consider other options. One participant noted:

I also think young people have more time [to live]. From what I understand, you can still have the surgery after the trial is over if the weight loss medication doesn’t work. (Participant 3, individual interview)

Being approved for bariatric surgery felt like passing through the eye of a needle, where participants perceived multiple points at which they risked being deemed unsuitable for the operation. As a result, they recommended that potential participants avoid losing their place on the bariatric waiting list or having to restart the entire process if the intensive weight loss program proves ineffective.

### The cost of weight loss medication was a major concern for participants

The cost of weight loss medications was a major concern for participants. The price of weight loss medications in Denmark is currently very high (ranging from 100 EUR to more than 300 EUR per month) and does not qualify for public reimbursement. Moreover, continuous treatment for an extended period is likely required [17]. Several participants were or had been on weight loss medication and found it to be a financial burden. Therefore, they considered it crucial for potential participants to know whether the medication would be provided free of charge during the trial:

If it costs anything [to participate] – that was my first thought [laughter] (…) because I know how expensive the medication is, right? (Participant 1, focus group)

The absence of public reimbursement for weight loss medication also makes bariatric surgery appear as the only option for those unable to afford long-term medication. For instance, a single mother of two had tried semaglutide for a few months before having to cease treatment:

It was expensive. It cost 1,800 [DKK per month] (approximately 240 EUR), to be more precise. So, it worked fine for the two months I could afford it. (Participant 1, individual interview)

She explicitly stated that if given the choice to participate in the clinical trial, she would decline, afraid that she would regain all her weight when the trial ended since she could not afford to pay for the medication on her own in the long term.

### Participants were concerned about the extra work involved in program participation

Most participants assessed the intensive weight loss program to be a more time-consuming option compared to bariatric surgery because it explicitly requires changes in diet and exercise habits—efforts they had attempted many times before without lasting success—and regular consultations with a dietitian. As one participant noted:

Many of us have already tried that, so it might be a bit of a letdown if one were to go through with it [the intensive weight loss program]. (Participant 2, focus group).

While the principles of maintaining a healthy lifestyle and adhering to medication may seem straightforward, the practical complexities—shaped by work, family, and lifestyle—can hinder the implementation of these habits in everyday life.

Participant 6: It is not complicated to get the injection once a week, but it is complicated with all the other things you have to coordinate in your everyday life (…).

Participant 8: But you could say that both [treatments] involve a lifestyle change in one way or another. You have to change your diet.

Participant 6: Yes, yes, that is true. (Focus group)

This exchange highlights the dynamic nature of focus group discussions, where the male participant's perspective contributed to a deeper understanding of the complexities involved in both treatment options. The discussion also addressed the time commitment required by the two treatment options concerning work absences. In Denmark, individuals undergoing bariatric surgery are recommended to take 3–4 weeks of paid medical leave post-surgery. This does not apply to those starting weight loss medication treatment, despite potential negative side effects. While the paid medical leave was seen as a benefit by some, one participant noted it was an additional burden on top of other potential healthcare issues, where weight loss medication could serve as an alternative:

Participant 4: There is also the perspective that if you already have sick leave due to back pain, coordinating additional leave with your employer can be difficult.

GO: Yes?

Participant 4: So in that case, the medication might be something you can use instead.

(Focus group)

On the other hand, concerns were raised about how employers might react if participants needed time off for dietary consultations as part of the intensive weight loss program:

There are many who have a job to consider, and it’s like, ‘I just need to take some time off,’ and then many employers start to count the costs, so to speak. (Participant 6, focus group)

Although the participants considered the IWL program as a more time-consuming option, none viewed the 104-week duration as a burden. The extra support was overall appreciated, however one participant did point out the need for flexibility in scheduling appointments with the dietician.

In summary, the discussion underscores the trade-offs associated with the two treatment options, emphasizing that both necessitate substantial time and effort. Balancing these demands alongside work and family responsibilities is a critical factor for individuals deciding between medical and surgical interventions for weight loss.

### Participants weighed the efficacy and potential negative side effects of surgery against those of an intensive non-surgical weight loss program based on personal beliefs and experiences

When reflecting upon being randomized to either medical or surgical weight loss treatment, it was important for participants how much and how fast weight loss would be obtained. Following the discussion above, it was believed that surgery would be the fastest way to lasting weight loss compared to an intensive non-surgical weight loss program.

Several participants indicated a preference for weight loss medication over surgery because they perceived it as less invasive:

Surgery is quite drastic. If the medication works, I would choose it over surgery any time.

Some participants found the irreversibility of surgery appealing. As one participant put it, surgery keeps you from “cheating” when feeling demotivated “because you no longer have a stomach.” In contrast, another participant likened weight loss medication to the treatment of alcoholism: “(…) [weight loss] medicine is a bit like Antabuse in the way that you just think, ‘To hell with it,’ you know, in some way, right?” (Participant 8, focus group). Indicating that with medication, you can choose to stop treatment at any time, unlike surgery, which is a permanent solution.

As mentioned previously, the interview took place in May/June 2023, 6 months after semaglutide was released in Denmark. The fact that the medication was new made some participants raise the question of whether they were part of a medical experiment testing potential side effects and adverse events of the new weight loss medication or part of a weight loss experiment with well-tested medication: “(…) you can say, well, how advanced is the research on that medicine? What do we actually know about it, what its long-term effects are, and all those kinds of things, right?” (Participant 8, focus group).

When discussing potential negative side effects from surgery, there was a tendency for participants to draw from previous experiences, arguing either for or against surgery. One participant, whose sister had undergone gastric bypass surgery and acquired serious side effects post-operation, said:

I’m actually not terribly worried about the [gastric sleeve], because that’s the one I’m getting [and not the bypass]. So, I’m not that concerned. The only thing I might fear a little is if I experience a lot of those side effects, but I don’t believe that will happen. There are no side effects with what I’m currently receiving [weight loss medication], so why would I get them for this? (Participant 2, individual interview)

Thus, the risks were counterbalanced by hope, both from the type of surgery and experiences with current treatment using medication. Another participant based her decision on an experience that happened years ago, making her hesitant to undergo future surgical procedures:

Well, I’m concerned about the actual surgery. I’m worried about the anesthesia, and I’m worried about the healing process. I was operated on and had a caesarean section when I gave birth to my daughter (…), and I developed an infection during the surgery. I contracted MRSA [a group of antibiotic-resistant bacteria], which prevented my caesarean scar from healing, so I had an open, oozing wound for 3 months. (…) Honestly, I would choose medical treatment; that’s what I would choose. (Participant 3, individual interview)

## Discussion

To understand and overcome potential obstacles in recruitment for a clinical trial (the LightBAR trial) comparing the effectiveness of bariatric surgery versus an intensive non-surgical weight loss program, a qualitative study was conducted. The findings illuminate the complexity of patient decisions in clinical trials where the treatment options are radically different. Patients who have experienced prolonged wait times for surgery exhibited a lower willingness to be randomized, reflecting their extensive history of weight loss attempts and the urgency to address their health concerns. Financial considerations were paramount, with the high cost of weight loss medications being a serious concern among participants. However, providing the medication for free, may to some extent alleviate these concerns. The practicalities of integrating treatment into daily life emerged as a critical factor, underscoring the need for interventions that fit patients’ lifestyles. Finally, patients mainly evaluated potential side effects and outcomes based on their own and others’ personal experiences. Based on these findings, the following changes were made to the trial to support the recruitment process: (a) potential trial participants are contacted immediately after referral to bariatric surgery, (b) it is emphasized both in the written information and verbally during consultations that the intensive non-surgical weight loss program, including total diet replacement and weight loss medication, is free of charge, and (c) it is clarified that both treatments require a substantial commitment of time and effort.

A limited number of studies have compared patients’ considerations regarding surgical or non-surgical lifestyle treatments. Strømmen et al. demonstrated that most patients favored bariatric surgery over a non-surgical lifestyle intervention due to their desire for a permanent solution. Having already tried various lifestyle-related approaches, they believed that surgery would prevent overeating. Additionally, work and family commitments hindered participation in the lifestyle intervention [18]. Motivations for a non-surgical lifestyle intervention among patients included a fear of anesthesia and complications, a desire for normality (believing they could lose weight through lifestyle changes and the right attitude), and a need for follow-up support to adapt to their new lifestyle. In contrast, Craig and colleagues found that 39% of their participants preferred nutritional therapy with support from medical professionals over bariatric surgery and pharmacotherapy alone. Cost, treatment accessibility, and negative side effects served as barriers to choosing pharmacotherapies and surgical therapies [19].

In our study, participants were concerned about the time and effort required to participate in the two interventions, such as changing lifestyle habits, absence from work, and clinical visits. Mattingly et al. coined the term “chronic homework” to describe the shift in treatment and care from clinics to home, which has resulted in additional responsibilities for patients and their families in managing chronic conditions [20]. When designing and presenting clinical trials, considering patients’ life circumstances and resources for carrying out this extra work is critical to ensure a fair representation of both interventions and align expectations, thereby avoiding future dropouts.

Our study suggests that recruitment for clinical trials that randomize bariatric surgery candidates should occur as early as possible, and ideally before patients are placed on the bariatric waiting list.

## Strengths and limitations

Obtaining access to patients who have been on the public waiting list for bariatric surgery and encouraging them to participate in a study in which they are asked to reflect on the possibility of randomization to surgery or an intensive non-surgical weight loss program is challenging due to the sensitivity of their situation and requires careful thought and planning.

We consider our study design, which incorporated vignettes during focus group discussions, as an advantage. The vignettes helped us create a safer environment for participants to share their views, as they were not required to speak from their own experiences or current life situations but rather from the perspective of fictional patients. Additionally, this approach allowed participants to reflect on randomization in relation to different patients and situations due to the variation in vignettes. This approach has also been effective in another study using a fictional case in focus groups on prostate cancer screening, which enabled participants to project their feelings onto the fictitious cases, facilitating more open and honest discussions about the risks, benefits, and decision-making processes [21].

Another strength of the study is the proportion of male participants, who make up almost half of the total, as they are usually underrepresented in weight loss studies, including bariatric surgery research [22].

However, the study does have certain limitations. Firstly, the limited number of participants recruited from a single hospital in the Capital Region of Denmark means that the sample may limit generalizability. Secondly, the difference in duration between the two focus groups, due to practical circumstances with expiring parking tickets, allowed for less elaboration of responses in the second focus group. Finally, the study participants spent significant time on the public waiting list for bariatric surgery, which could have biased the results; eight out of nine expressed a strong desire towards getting bariatric surgery. However, since the participants would not be invited to participate in the clinical trial, a preference for bariatric surgery was expected

## Conclusions

The study highlights key concerns patients have when considering enrolment in an RCT comparing bariatric surgery with an intensive non-surgical weight loss intervention. Important topics include the decision to undergo irreversible surgery, the financial implications, the extra work involved in participation, and the potential health benefits and harms of each option. These findings are expected to aid in designing approaches to participant recruitment in future clinical trials, particularly those evaluating fundamentally diverse treatment modalities in weight loss management. This could involve addressing existential themes in the recruitment strategy and ensuring that patients are given sufficient time to make decisions about treatments that may be life changing.

## Supplementary Information

Additional file 1. Vignettes.

Additional file 2. Focus group moderator guide.

Additional file 3. Bariatric surgery waiting list interview guide.

Additional file 4. Consolidated criteria for reporting qualitative studies (COREQ): 32-item checklist.

## Data Availability

Due to the sensitive nature of our data, sharing it publicly is not feasible as it could compromise privacy. However, we substantiate our claims in the article through relevant quotations.

## Abbreviations

DKK: The Danish krone
EUR: Euro
RCT: Randomized controlled trial
