# Supplementary material for: Time, money, and weight loss: a qualitative study exploring patients’ perspectives on randomization for bariatric surgery vs. an intensive non-surgical weight loss program
Source: Trials. 2025 Apr 4;26:121. doi: 10.1186/s13063-025-08816-8 (PMC11971855; doi:10.1186/s13063-025-08816-8)
Supplement: Supplementary file 1 — Additional file 1. Vignettes. [file 13063_2025_8816_MOESM1_ESM.docx]

**Additional file 1**

**Vignettes**

Mette, a 46-year-old mother of two, works in a store. She commutes for an hour to and from work each day and walks the family dog in the morning. She often experiences back pain but is generally healthy.

Peter, 60 years old, works in an office and has lived alone since his divorce. He has three grown-up children, two of whom he sees frequently. He wants to play football with his two younger sons. He has high blood pressure but is otherwise healthy.


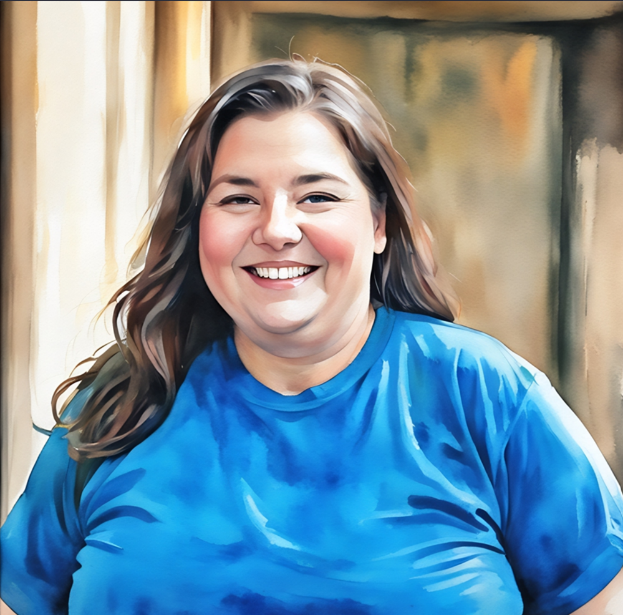

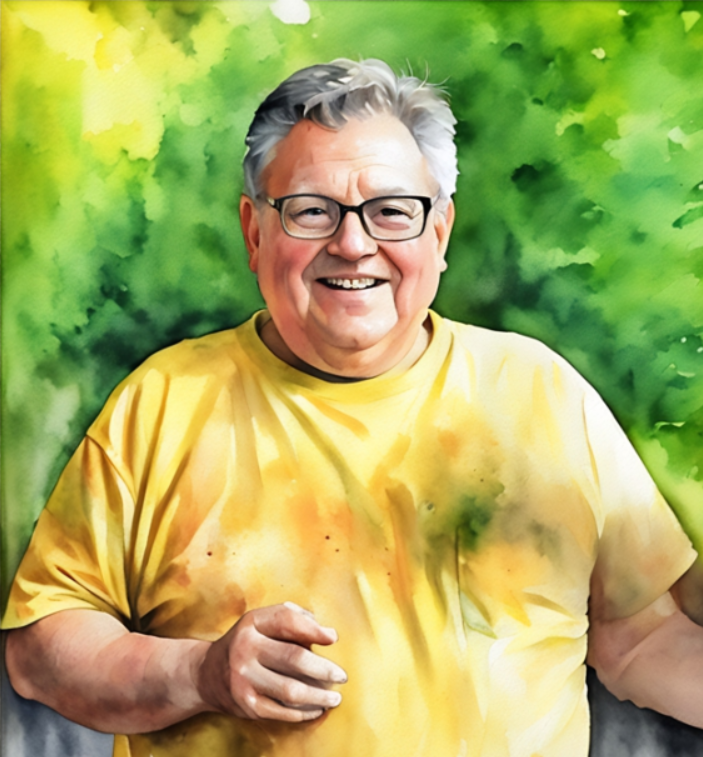


Pictures powered by OpenArt.ai and edited with BeFunky.com.
