# Supplementary material for: Time, money, and weight loss: a qualitative study exploring patients’ perspectives on randomization for bariatric surgery vs. an intensive non-surgical weight loss program
Source: Trials. 2025 Apr 4;26:121. doi: 10.1186/s13063-025-08816-8 (PMC11971855; doi:10.1186/s13063-025-08816-8)
Supplement: Supplementary file 2 — Additional file 2. Focus group moderator guide. [file 13063_2025_8816_MOESM2_ESM.docx]

**Additional file 2**

**Focus group moderator guide**

**Introduction:**

- Thank you for your participation.
- Aim and duration of the interview: To gather your perspectives on what is important for patients. The interview takes about an hour.
- Explain the different roles of the researchers.
- CD explains the trial and the research project.
- Participants introduce themselves (First name, age, children, occupation).

**Vignette:**

- Moderator (GO) reads vignettes aloud.
- GO explains: “Imagine that Mette and Peter are invited to be a part of the trial.”
- How do you think they would prefer to be invited for the trial?
- What is important for them to know before they potentially say yes?
- What could make them withdraw from the trial?
- What could researchers do to prevent them from withdrawing?

**Thank you and end of interview.**
