# Supplementary material for: Time, money, and weight loss: a qualitative study exploring patients’ perspectives on randomization for bariatric surgery vs. an intensive non-surgical weight loss program
Source: Trials. 2025 Apr 4;26:121. doi: 10.1186/s13063-025-08816-8 (PMC11971855; doi:10.1186/s13063-025-08816-8)
Supplement: Supplementary file 3 — Additional file 3. Bariatric surgery waiting list interview guide. [file 13063_2025_8816_MOESM3_ESM.docx]

**Additional file 3**

**Bariatric surgery waiting list interview guide**

**Introduction:**

- Purpose: We are interested in investigating two significantly different treatments (weight loss medication vs. bariatric surgery), and to do so, it's crucial to understand how to engage people in such a trial effectively, ensuring it aligns with their needs.
- Therefore, we're interested in hearing what you hope for or are concerned about regarding these treatments.

**Follow-up on focus group interview:**

- Have you reflected on anything from the focus group interview that you'd like to delve further into?

**Background information:**

- Could you please share your name, age, and where you're from?
- What is your typical daily routine?
- Have you previously tried weight loss medication, similar to the one utilized in this study?
- How long have you been on the bariatric surgery waiting list?

**Others’ experiences:**

- Do you know someone who has undergone bariatric surgery?
- Do you know someone who has used weight loss medication?
- What are their experiences?

**Perceptions of weight loss medication:**

- What outcomes do you anticipate if you were to take the medication?
- What concerns do you have about taking the medication?

**Perceptions of bariatric surgery:**

- What outcomes do you expect after undergoing the surgery?
- What concerns do you have about having the surgery?

**General fatigue and exhaustion (symptoms after both):**

Many individuals experience fatigue and discomfort when they consume insufficient calories.

- How do you typically feel when you haven't consumed enough calories?

**Difference between the two treatments:**

What distinguishes these treatments significantly is that medication can be discontinued, whereas surgery is irreversible.

- Do you perceive it as advantageous that one treatment is more permanent than the other?
- If you had the choice, which treatment would you opt for? Why?
- Which treatment do you think most people would prefer, given the choice? Why?

**Life after surgery:**

- Following surgery, how do you envision utilizing your body in your daily activities?
- What are you most eager to achieve by undergoing bariatric surgery?
- Are there any activities or experiences you're looking forward to trying?

**Randomization (preconception, perceptions):**

- Why do you believe researchers consider it essential to randomly assign participants to different treatments and then remain in their assigned group?
- How would you feel about undergoing surgery after your assignment has been determined randomly?
- Do you think receiving treatment as part of a trial would differ from being on a waiting list? If so, explain how and why?
- In the event of being randomized to the medication group, what factors might lead you to discontinue participation?

**Final remarks and thank you:**

- Is there anything I haven't addressed that you'd like to discuss?
- What key takeaway would you like me to remember from our interview?
- Please don't hesitate to reach out if you have any additional thoughts or questions.
